# Metasynthesis: An Original Method to Synthesize Qualitative Literature in Psychiatry

**DOI:** 10.3389/fpsyt.2017.00269

**Published:** 2017-12-01

**Authors:** Jonathan Lachal, Anne Revah-Levy, Massimiliano Orri, Marie Rose Moro

**Affiliations:** ^1^AP-HP, Cochin Hospital, Maison de Solenn, Paris, France; ^2^Université Paris Descartes, Sorbonne Paris Cité, Paris, France; ^3^CESP, Faculté de médecine, Université Paris-Sud, Faculté de médecine, Université de Versailles Saint-Quentin-en-Yvelines (UVSQ), INSERM, Université Paris-Saclay, Villejuif, France; ^4^Service Universitaire de Psychiatrie de l’Adolescent, Centre Hospitalier Argenteuil, Argenteuil, France; ^5^ECSTRA Team, UMR-1153, INSERM, Paris Diderot University, Sorbonne Paris Cité, Paris, France; ^6^Université Paris-Sud, Paris, France

**Keywords:** qualitative research, metasynthesis, metaethnography, qualitative evidence synthesis, psychiatry, suicide

## Abstract

**Background:**

Metasynthesis—the systematic review and integration of findings from qualitative studies—is an emerging technique in medical research that can use many different methods. Nevertheless, the method must be appropriate to the specific scientific field in which it is used. The objective is to describe the steps of a metasynthesis method adapted from *Thematic Synthesis* and phenomenology to fit the particularities of psychiatric research.

**Method:**

We detail each step of the method used in a metasynthesis published in 2015 on adolescent and young adults suicidal behaviors. We provide clarifications in several methodological points using the latest literature on metasyntheses. The method is described in six steps: define the research question and the inclusion criteria, select the studies, assess their quality, extract and present the formal data, analyze the data, and express the synthesis.

**Conclusion:**

Metasyntheses offer an appropriate balance between an objective framework, a rigorously scientific approach to data analysis and the necessary contribution of the researcher’s subjectivity in the construction of the final work. They propose a third level of comprehension and interpretation that brings original insights, improve the global understanding in psychiatry, and propose immediate therapeutic implications. They should be included in the psychiatric common research toolkit to become better recognized by clinicians and mental health professionals.

## Background

The use of qualitative research is proliferating in medical research (1). Over the past two decades, numerous studies in the field of psychiatry have used a qualitative protocol (2, 3), and it has been recognized as a valuable way to “*obtain knowledge that might not be accessible by other methods and to provide extensive data on how people interpret and act upon their illness symptoms*” (4). What matters most is the respondent’s perspective and the joint construction by the respondent and the researcher of a context-dependent, multiple, and complex reality (5). In this respect, the qualitative approach is close to that of the psychiatrist: what is important is what the patient feels and experiences and what emerges during the interaction between the patient and the psychiatrist. Indeed, the subjective coconstruction inherent to most of qualitative methods seems especially close to the psychiatric clinical meeting. Both are useful for building up local theory that helps to increase two important aspects of theory: individually relevant theory for clinical work and field-specific general theory for research (6). Qualitative research offers a thick description (one that encompass all the complexity of the phenomenon, behavior, or context) of a phenomenon and attempts to document the complexity and multiplicity of its experience (6). Similarly, in their day-to-day clinical work, psychiatrists attribute great importance to complexity and try to place symptoms within the patient’s history, in all of its intricate context—which again plays a crucial role in therapeutic choices.

Some have expressed concern, however, that because qualitative studies are isolated and rarely used to contribute to practical knowledge, they do not play a significant role in the movement toward evidence-based medicine (5). To alleviate this concern and enable qualitative work to contribute to this movement, an increasing number of teams have worked to develop and apply synthesis methods to these data. Qualitative syntheses refer to a collection of different methods for systematically reviewing and integrating findings from qualitative studies (7). The aims of such methods are to capture the increasing volume of qualitative research, to facilitate the transfer of knowledge to improve healthcare, and to bring together a broad range of participants and descriptions (8, 9). Qualitative syntheses require not only a systematic approach to collecting, analyzing, and interpreting results across multiple studies, but also to develop overarching interpretation emerging from the joint interpretation of the primary studies included in the synthesis (10, 11). Therefore, it involves going beyond the findings of any individual study to make the “whole into something more than the parts alone imply” (12).

Qualitative syntheses are now recognized as valuable tools for examining participants’ meanings, experiences, and perspectives, both deeply (because of the qualitative approach) and broadly (because of the integration of studies from different healthcare contexts and participants). They have been shown to be particularly useful to identify research gaps, to inform the development of primary studies, and to provide evidence for the development, implementation, and evaluation of health interventions (13). Because of this growing importance, an important work has been done in the last ten years, in order to ensure the quality of qualitative syntheses, such as: describing the methods to ensure reproducibility, develop tools for assessing the quality of the primary articles, and establish reporting guidelines [see, for example, the ENTREQ statement (13), the GRADE-Cerqual protocol (14), and the Cochrane or EVIDENT works (15, 16)].

**Figure 1:**
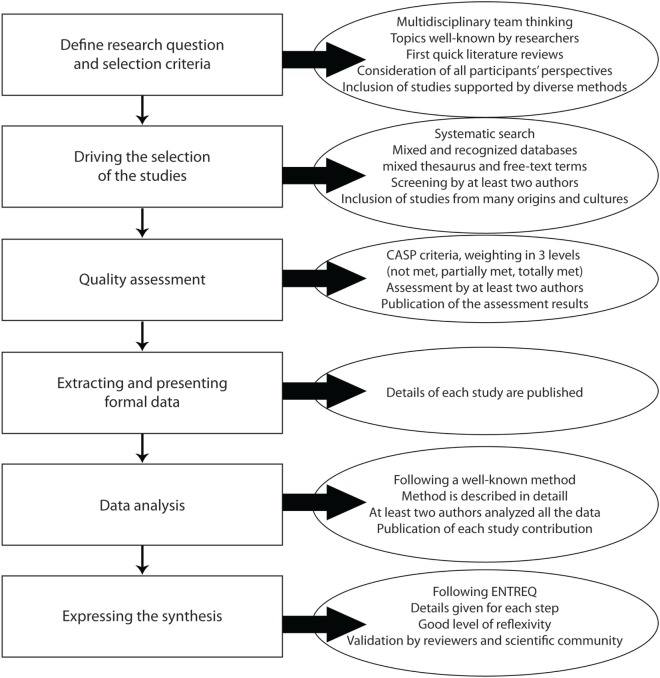
Distribution in time for articles included in the metasynthesis.

However, despite some qualitative syntheses have been successfully conducted in the field of mental health (2, 3, 17–20), no study considers the methodological specificities inherent to psychiatric epistemological stance (7). Filling this gap has been one of the aims of our team since 2011. In this methodological article, we aimed to discuss the challenge of implementing metasynthesis to improve the understanding of youths suicide. In this study, we adapted the Thematic Synthesis developed by Thomas and Harden and incorporate a phenomenological approach in order to deal with new rigor with general as well as psychiatric issues (21). We will present each step of the method (Figure 1) and will propose methodological discussions. The detailed description of the findings can be found elsewhere (22).

## Conducting a Metasynthesis

### Before Start—Constitution of a Research Group

The constitution of the research group and the definition of the study method are an important step before engaging in any synthesis work. The researcher must work in collaboration with researchers of diverse backgrounds (9). A collaborative approach improves quality and rigor and subjects the analytical process to group reflexivity (11). The research team should include members trained in qualitative synthesis as well as those expert in the topic being studied (23). As there are many ways to do qualitative syntheses, the research team will have to choose one of them adapted to the research question and to the expertise of the group (15).

Our team is composed of adolescent and child psychiatrists and psychologists from France and elsewhere (Italy, Chile, and Brazil) and focuses on developing qualitative research (24–26) and metasynthesis in adolescent psychiatry and related fields (22, 27, 28). Our method is adapted from thematic synthesis (21), which combines and adapts approaches from both metaethnography and grounded theory (10). Metaethnography, as well as Thematic Synthesis, takes place in six or seven steps from data collection to text coding and finally writing the synthesis. Original authors of metaethnography were trained in grounded theory, a qualitative method developed in the social sciences, laying on conceptual coding combine to construct a new theory. Thematic synthesis allows the researcher to include much more studies in the synthesis and to use tools coming from quantitative reviews, as systematic literature searches. This method perfectly suits to psychiatric research: user-friendliness for both researchers and readers; standardized in its most subjective steps but flexible, to make it adaptable to various patients or situations, such as children, patients with psychological disabilities or psychotic disorders, and to different researchers’ backgrounds (e.g., phenomenology, psychology, or psychoanalysis). We add a phenomenological perspective with a coding close to Smith’s interpretative phenomenological analysis (IPA) (29). IPA is also a qualitative method of coding a text, laying on phenomenology and hermeneutics. The level of coding is what makes sense to the reader (for example, a letter, a word, a sentence, the absence of a word, or a sentence). Phenomenology allows avoiding never-ending debates about theories of the psyche and focuses on the patient experience which is at the heart of psychiatric care. We understand that published manuscripts provide only thin data sets that are not eligible for a complete phenomenological analysis. Rather we tried to let ourselves guided by the impressions the text generated in us. It was like one article was assimilated as one participant, as it is mainly the voice of the main writer. We applied Smith’s tips on how reading and coding the data.

### Define the Research Question and the Selection Criteria

Defining the research question is a crucial substep (9). This question must be broad enough to be of interest but small enough to be manageable (5, 23) and has already been explored by enough studies (30). Inclusion and exclusion criteria may be fixed on methodological aspects, on participants selected, on thematic focuses or language specificities (9, 31).

Youths suicide is a focus that were suitable for qualitative methods. We chose this subject because youth suicide is a major public health issue worldwide as well as a complex disorder that encompasses medical, sociological, anthropological, cultural, psychological, and philosophical issues. It has been widely explored by qualitative research. The lack of effectiveness of current care let us think that new insights could be expected by qualitative exploration. A first selection of articles, as well as an existing literature review on the topic, served to specify some starting information and enable initial decisions, including the definition of the research question, specification of the scope and the inclusion criteria. Then, the questions were constructed through reading and confronting these articles with our first qualitative study in the theme and our clinical knowledge of the theme.

As we wanted to study the therapeutic relationship and barriers to effective care, we decided to include research concerning not only the population being treated (the adolescents and young adults, and their parents), but also the healthcare professionals who care for these patients. A first screening of the literature showed us that optimal scope required a large range of ages, from 15 to 30 years old. The common thread linking all these youths was the importance of their parents in their everyday life. We chose to include only qualitative research, because it remains unclear how to deal with mixed method (combining qualitative and quantitative datasets) (23). Although databases contain articles in different languages, we chose to include only articles published in English (as most studies are now published in English) and French (as it is our first language) (22, 27).

### Study Selection

There is a debate on the choice of sampling method, some authors using an exhaustive sampling, some others, an expansive one (30). We privileged exhaustive systematic searches (32) since our method allowed large samples and because our target audience was the mental health community, which is accustomed to quantitative systematic reviews (9). Only journal articles were included, as most scientific data are published in this form (33). The first selection of articles served to specify the choice of keywords and databases for the electronic search. To ensure both sensitivity and specificity, we decided to use a combined approach of thesaurus terms and free-text terms. This technique maximizes the number of potentially relevant articles retrieved and ensures the highest level of rigor (34). Keywords were established during research team meetings, and were reported in the article or as supplemental material for more clarity (35). As each database has its own thesaurus terms, and as keywords encompasses different meanings in each discipline (36), the keywords were specific for each one.

We used four clusters of keywords: (i) those that concern the topic of interest (such as suicide, obesity, or anorexia nervosa), (ii) those that concern the participants (gender, age, profession, etc.), (iii) those that concern qualitative research (such as *qualitative research, interviews, focus groups*, or *content analysis*), and (iv) those that concern perceptions and understanding, often called “views” (33) (such as *knowledge, perception, self-concept, feeling*, or *attitude*). The last cluster takes all its importance in the phenomenological perspective of the analysis. An example of the final algorithm used (in the PubMed Web search) is provided in Table 1.

**Table 1:** Algorithm used in the PubMed Web search from Ref. (22).

((MH “Suicide+”) OR (MH “Suicidal Ideation”) OR (MH “Suicide, Attempted”) OR (“suicide Attempts”) OR (“suicide”) OR (“attempted suicide”) OR (“suicidal ideation”) OR (“suicide ideation”) OR (“suicidal behavior”) OR (“youth suicide”) OR (MH “Self mutilation”) OR (MH “Self-Injurious Behavior+”) OR (“overdose”) OR (“self poison*”) OR (“self inflict*”) OR (“self harm*”) OR (“self cut*”) OR (“self destruct*”) OR (“self-injury*”) OR (“self mutilate*”))
AND
((MH “Adolescent”) OR (MH “Young Adult”) OR (MH “Adolescent Psychology”) OR (MH “Adolescent Psychiatry”) OR (MH “Adolescent Behavior”) OR (MH “Adolescent Development”) OR (“teenagers”) OR (“teens”) OR (“adolescence”) OR (“adolescent”) OR (“adolescents”) OR (“young adult”) OR (“young”))
AND
((MH “Qualitative research”) OR (MH “Nursing Methodology Research”) OR (MH “Focus Groups”) OR (MH “Observation”) OR (“qualitative research”) OR (“qualitative study”) OR (“qualitative method”))
AND
((MH “Knowledge”)OR (MH “Psychology”) OR (MH “Self Concept”) OR (MH “Adolescent Psychiatry”) OR (MH “Attitude”) OR (MH “Perception”) OR (MH “Self Concept”) OR (“perception”) OR (“attitude”) OR (“feeling”) OR (“knowledge”) OR (“belief”) OR (“view”) OR (“perspective”) OR (“opinion”) OR (“experience”) OR (“image”) OR (“self concept”) OR (“barrier*”) OR (“psycholog*”) OR (“psychiatry”))

Similar work was conducted to select the databases. After consulting reference articles (33, 37, 38), we decided to conduct the search in five electronic databases covering medical, psychological, social, and nursing sciences: MEDLINE, EMBASE, CINAHL, PsycINFO, and Social Science Citation Index (SSCI). Not long ago, CINAHL was the most important database for finding qualitative research, but as qualitative research proliferates in medical research, more and more qualitative articles are referenced in MEDLINE (33) and EMBASE. PsycINFO was a good database for finding qualitative articles with a psychological approach. We decided to add SSCI to broaden and complexity the outlook with a sociological point of view. We followed recommendations published on MEDLINE (39), CINAHL (40), EMBASE (41), and PsycINFO (42) for choosing search terms. Finally, we decided not to use the methodological databases’ filters for qualitative research, as these have undergone little replication and validation (43).

**Figure 2:**
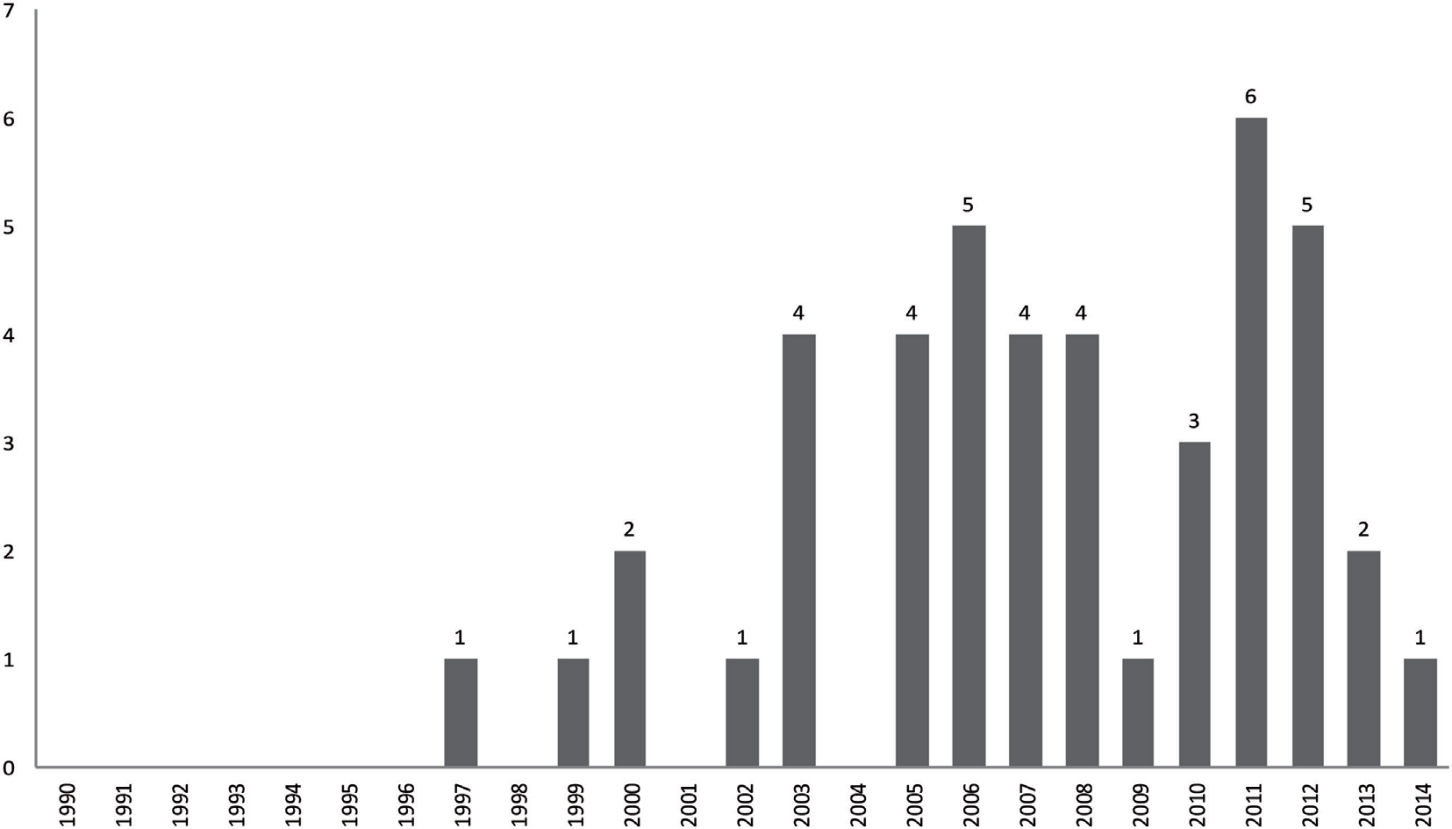
Flowchart of the metasynthesis steps.

We decided to include articles published only in or after 1990. Two points impelled this decision: first, there was very little qualitative research on suicide before the year 2000 and even less before the 1990s (Figure 2). Second, we chose to consider as outdated research findings and results published more than 20 years ago were outdated, given the evolution of medical practices (44). However, this choice must be adapted to the topic of metasynthesis.

**Figure 3:**
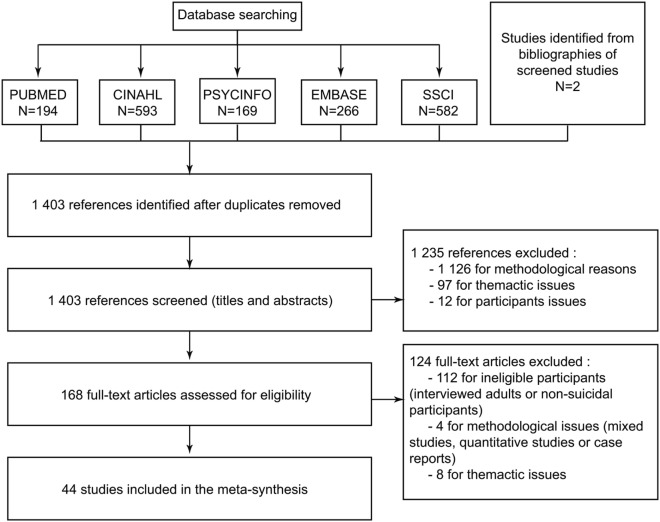
Flowchart for selecting studies from Ref. (22).

The results of database searches were entered into a bibliographic software program (Zotero©) for automatic removal of duplicates. Then, two authors independently screened all titles and abstracts and selected the studies according to our inclusion criteria (defined earlier). If the abstract was not sufficient, we read the full text. Disagreements were resolved during working group meetings. Full texts of potentially relevant articles were then examined, and a second selection was performed. At this phase, we also checked each article’s reference list looking for new articles we might have overlooked. The final selection represented from 2 to 3% of the total initially obtained. This rate is consistent with the findings of other metasyntheses (23). For clarity, the selection process was also presented in a flowchart (Figure 3). We referred to STARLITE principles to report our literature search (45) (Table 2).

**Table 2:** STARLITE principles applied to the literature search report of Ref. (22).

#	Criteria	Result	# in the original publication
S	Sampling strategy	Comprehensive	p. 3
T	Type of Study	Fully reported (any kind of qualitative study)	p. 3
A	Approaches	Electronic and citation snowballing	pp. 3–4
R	Range of years	Fully reported 01-1990 until 05-2014	p. 3
L	Limits	Language (English and French)	p. 3
I	Inclusion and exclusions	Inclusion (qualitative method, specifically concerned suicidal behaviors in adolescents and young adults, interviewed young people who were suicidal, or who had attempted suicide in their youth, or parents of these youth, or medical professionals who provide care to suicidal youth). Exclusion (quantitative or mixed methods; studies in the general population exploring prevention of suicide or social representations of suicide in adolescents and young adults; studies concerning solely deliberate self-harm or non-suicidal self-injury)	p. 3
T	Terms used	Complete search strategy published in Online Supplemental Data	S2 Table
E	Electronic sources	MEDLINE, EMBASE, CINAHL, SSCI	pp. 3–4

### Quality Assessment of Included Studies

There is no consensus about whether quality criteria should be applied to qualitative research, or, for those who think they should be, about which criteria to use and how to apply them. Nevertheless a growing number of researchers are choosing to appraise studies for metasyntheses (46) and some authors state that a good metasynthesis can no longer avoid this methodological step (7). The reasons and methods for quality assessment fit into three general approaches: assessment of study conduct, appraisal of study reporting, and an implicit judgment of the content and utility of the findings for theory development (13). There is certainly not one best appraisal tool, but rather a wide choice of good ones (8).

We chose the Critical Appraisal Skills Program (CASP) (47), which is the most frequently used instrument (46), addresses all the principles and assumptions underpinning qualitative research (13). It is one of the instruments recommended by the Cochrane Collaboration (48) and has been used in several important thematic analyses of medical topics. As proposed by Boeije et al., we weighted our assessment by applying a three-point scale to each criterion (0 = criterion not met; 1/P = criterion partially met; 2/T = criterion totally met) (49) (Table 3).

**Table 3:** Evaluation of the quality of the studies according to the Critical Appraisal Skill Programme (CASP) from Ref. (22).

Criteria	Totally met^a^	Partially met^a^	Not met^a^
1. Was there a clear statement of the aims of the research?	41	3	0
2. Is a qualitative methodology appropriate?	41	3	0
3. Was the research design appropriate to address the aims of the research?	39	5	0
4. Was the recruitment strategy appropriate to the aims of the research?	31	10	3
5. Were the data collected in a way that addressed the research issue?	37	6	1
6. Has the relationship between researcher and participants been adequately considered?	25	10	9
7. Have ethical issues been taken into consideration?	36	1	7
8. Was the data analysis sufficiently rigorous?	24	16	4
9. Is there a clear statement of findings?	28	9	7
10. How valuable is the research?	29	15	0

*^a^Number of studies*.

We have not excluded any study on quality criteria. We think that the goal of the quality assessment is not to help selecting the more rigorous article. Either, this step is important to improve the overall rigor of the metasynthesis: by easily evaluating the quality of each article, the readers will have the possibility to make their own evaluation of the quality of the results of the metasynthesis (9). To enhance the rigor of the synthesis, we published the full results of this assessment (50).

### Extracting and Presenting the Formal Data

To understand the context of each study, readers need the formal data about each study: the number and type of participants in each study, its location, and the method of data collection and of analysis. These data must be extracted and presented in a way that enables readers to form their own opinions about the studies included. We presented these data systematically, in a table with the following headings:

– Identification of the study.
– Summary of the study’s aim.
– Country where the study took place.
– Details about the participants: age, gender, type, and number.
– Method of data collection (e.g., semistructured interviews or focus groups).
– Analysis method (grounded theory, phenomenology, thematic, etc.).

### Data Analysis

This step is probably the most subjective: its performance is highly influenced by the authors’ backgrounds (13). There are many ways to analyze, as many as there are authors. All researchers build on their personal knowledge and background for the analysis, sometimes described as *bricolage*, following Claude Levi-Strauss: “*the* bricoleur *combines techniques, methods, and materials to work on any number of projects and creations. Whereas a typical construction process might be limited by the history or original use of individual pieces, the* bricoleur *works outside of such limitations, reorganizing pieces to construct new meaning. In other words, unlike linear, step-by-step processes, the* bricoleur *steps back and works without exhaustive preliminary specifications*” (51, 52). The synthesis will inevitably be only one possible interpretation of the data (9), as it depends on the authors’ judgment and insights (21). The qualitative synthesis does not result simply from a coding process, but rather from the researchers’ configuration of segments of coded data “*assembled into a novel whole*” (53).

In this process, the multidisciplinary team is essential to assess rigor and develop richer and more complex understandings. Triangulation of the analyses is enhanced when researchers with diverse background consider the same data set (11). “*Collaborative working not only improve quality and rigour, but subjects the analytical process to group reflexivity*” (54).

The first step of this process involved carefully reading and rereading each study (21). It is an active reading, with the intention of appraising, familiarizing, identifying, extracting, recording, organizing, comparing, relating, mapping, stimulating and verifying. In other words, it is reading with “*the intention of collating a synthesizable set of accounts*” (11).

The second step was coding: at least two different researchers coded each part of the data (whole manuscripts), performing a line-by-line coding, close to the phenomenological analysis described by Smith et al. (29).

In the third step, the codes were grouped and categorized into a hierarchical tree structure. This step is very close to the translation work described by Noblit and Hare (12). It involves comparing themes across articles to match themes from one article with those from another while ensuring that each key theme captured similar themes from different articles. We obtained a list of descriptive themes very close to the data.

In our example, we highlighted the sentence “You’re going to school, you’re getting an education, but you’re depressed” and coded it *depressed*. The code is then combined with others in a theme named “The experience of distress.”

Finally, in the last and most subjective step of the analysis, we generated analytical themes, which depended largely on the “judgment and insights of the reviewers” (21). This step is very similar the development of third-order interpretations—“*the synthesis of both first and second order constructs into a new model or theory about a phenomenon*” (23)—and requires going beyond the content of original studies to achieve a higher level of interpretation and going beyond the descriptive synthesis to propose a more conceptual line-of-argument (21, 23). This work has two types of underlying aims. The first type may be theoretical, by enabling a higher level of comprehension of a phenomenon; in medical science, this may be to better describe and understand a pathology. The second type may be to answer clinical questions about pathology and care directly.

In our example, we clearly fulfilled the second aim. The results leaded us to discuss new insights about suicidal youths’ care. The experience of incomprehension shared by all the protagonists of the care interferes with the capacity for empathy of both family members and professionals. We could use the concepts of intersubjectivity to witness the violence driven by the suicidal act.

### Writing the Synthesis

Throughout the analysis process, the authors build themes that take place in the *story* they are telling about the participant’s experience (21). Then, the expression of the synthesis is our story of the studied phenomenon.

The results of the metasynthesis consist of the themes that we developed in the analysis. They are built by first and second order constructs. We did not define actual third-order themes; rather, third-order constructs helped us to build the synthesis into a story. We organized the themes into superordinate themes, which are interpretations of the themes and can be considered third-order interpretations.

For example, in one of the developed theme called *the experience of distress* we described that the young people experienced depressive symptoms. The participants described feelings of *sadness, sorrow, mental pain, despair, detachment, anger, and irritability*. The authors interpreted that as *despair*. We organized all these closed related feeling into the *individual experience of distress*. We decide not to speak about *depression*, first because some healthcare professionals repeated that they may diagnose depression “*but certainly not on a routine basis*” (22), then because we adopted a phenomenological approach and we felt that *distress* encompasses a broader and more complex experience.

Metasynthesis results prepare the framework for the discussion, the most interpretative part of the review, where hypothesis and proposals are presented. We offer our understanding of the participants’ experience. Both our presentation and our discourse are influenced by our aim: to answer clinical questions by suggesting specific actions or considerations for care; the discussion and the answers are intended to be useful for the readers of our article, as well as for us (23).

Our conclusion is that “*the violence of the message of a suicidal act and the fears associated with death lead to incomprehension and interfere with the capacity for empathy of both family members and professionals. The issue in treatment is to be able to witness this violence so that the patient feels understood and heard, and thus to limit recurrences*” (22). This issue is clear and simple and it leads to an immediate application to clinical practice which is described in the implication for practice chapter.

Finally, we discuss the limitation of the findings. The principal limitations were methodological (with our method, the access to participants’ data is partial), and in the sampling (we didn’t take in consideration the influence of gender on the experience of suicidal behavior). That exercise enhances the credibility of the publication, enabling readers to measure the importance and generalizability of the findings.

The written synthesis has to fulfill the standard for reporting synthesis of qualitative research. We chose to refer to Tong and al. ENTREQ statement (13) attached to the publication.

## Conclusion

Our method is based on Thomas and Harden *Thematic Synthesis* (21). After a broad-scale review of literature on the topic of metasynthesis, we have decided to clarify the definition of some aspects of the method and modify or expand others, because we wanted both a medical and a psychological approach. For example, we opted to use a systematic search method and a weighted version of the CASP to assess quality.

Most metasynthesis authors argue that these reviews achieve a third-order level of interpretation, that is, that they are more than the sum of their results. If, as we think, qualitative research can achieve a moderate level of generalization with clinical implications, metasyntheses may transform these findings into more highly abstracted and generalizable theoretical frameworks. We “*push their findings toward the nomothetic end of the idiographic-nomothetic continuum*” (44). Qualitative specialists certainly do not shy away from stressing the importance of context in their studies, or from arguing that the context of one study may not be applicable to others. It is true that, in a way, metasyntheses decontextualize concepts to attain greater generalizability (44). But we can relate this act to the response of clinicians reading a qualitative article: they will try to apply the concepts to their own situations (21). Authors of metasyntheses are proposing their own interpretation of the concept and its generalizability. The scientific value of metasynthesis lies in its role as a summary of several studies and as the interpretation of varying context, as well as in its ability to weight each result and to propose greater generalizability.

Qualitative research is an invaluable method for gaining new insights into mental disorders (6). Its development in recent years requires that we improve methods for synthesizing their results. We think this way of doing metasynthesis is appropriate to psychiatric research in its intermediate position that stresses both progress in the general comprehension of disorders and direct clinical implications. It offers an appropriate balance between three components: an objective framework, which includes the selection, inclusion, and appraisal of studies; a rigorously scientific approach to data analysis; and the necessary contribution of the researcher’s subjectivity in the construction of the final work. The balance for a qualitative metasynthesis is, we think, very similar to the clinical approach to each patient. It necessitates a robust scientific background, a rigorous step-by-step—symptom by symptom—progression, and finally a part of *art* that depends on each clinician: the subjective part of therapy.

Finally, we think that metasyntheses enable insights that no other method can provide. Qualitative research sheds new light on scientific questions by emphasizing the participants’ subjective understanding and experience (6). Metasynthesis proposes a third level of comprehension and interpretation that brings original insights. In our study (22), we emphasized an original point in the relationship that was no found in any result from each primary study: the difficulty of professionals and parents to understand and cope with suicide as an obstacle to the care of the suicidal adolescent. Therefore, our study’s analysis went deeper and proposed original results.

## Author Contributions

Conceived and designed the experiments and final approval: JL, AR-L, MO, and MM. Conducted the literature review: JL and MO. Performed the experiments: JL, MO, and AR-L. Wrote the article: JL (all the article), AR-L (analysis), MO (introduction and analysis), and MM (discussion).

## Conflict of Interest Statement

The authors declare that the research was conducted in the absence of any commercial or financial relationships that could be construed as a potential conflict of interest.
